# Predictors of Treatment Adherence and Virological Failure Among People Living with HIV Receiving Antiretroviral Therapy in a South African Rural Community: A Sub-study of the ITREMA Randomised Clinical Trial

**DOI:** 10.1007/s10461-023-04103-2

**Published:** 2023-06-29

**Authors:** Siphamandla B. Gumede, Annemarie M. J. Wensing, Samanta T. Lalla-Edward, John B. F. de Wit, W. D. Francois Venter, Hugo A. Tempelman, Lucas E. Hermans

**Affiliations:** 1 Ezintsha, Faculty of Health Sciences, University of the Witwatersrand, 32 Princess of Wales Terrace, Parktown, Johannesburg, 2193 South Africa; 2 Department of Interdisciplinary Social Science, Utrecht University, Utrecht, The Netherlands; 3 Department of Medical Microbiology, University Medical Center Utrecht, Utrecht, The Netherlands; 4 Ndlovu Research Consortium, Elandsdoorn, South Africa; 5 Centre for Social Research in Health, UNSW, Sydney, Australia

**Keywords:** Antiretroviral therapy, Adherence, Pill count, Virological failure, Depression, South Africa

## Abstract

A large proportion of people living with HIV (PLHIV) in sub-Saharan Africa reside in rural areas. Knowledge of enablers and barriers of adherence to antiretroviral treatment (ART) in these populations is limited. We conducted a cohort study of 501 adult PLHIV on ART at a rural South African treatment facility as a sub-study of a clinical trial (ClinicalTrials.gov NCT03357588). Socio-economic, psychosocial and behavioral characteristics were assessed as covariates of self-reported adherence difficulties, suboptimal pill count adherence and virological failure during 96 weeks of follow-up. Male gender was an independent risk factor for all outcomes. Food insecurity was associated with virological failure in males. Depressive symptoms were independently associated with virological failure in both males and females. Household income and task-oriented coping score were protective against suboptimal pill-count adherence. These results underscore the impact of low household income, food insecurity and depression on outcomes of ART in rural settings and confirm other previously described risk factors. Recognition of these factors and targeted adherence support strategies may improve patient health and treatment outcomes.

**Supplementary Information:**

The online version contains supplementary material available at 10.1007/s10461-023-04103-2.

## Introduction

South Africa (SA) has an estimated HIV prevalence of 13.7%, with approximately 8.2 million people living with HIV (PLHIV) in 2021 [1]. Over one-third of the South African population resides in rural settings [2]. Rural populations are characterized by disadvantaged socio-economic status, limited access to healthcare services, and poor infrastructure and healthcare resources, when compared to their urban counterparts [3]. These factors adversely affect access and adherence to HIV treatment, potentially resulting in worse health outcomes for PLHIV [3, 4].

Studies report episodes of ART non-adherence in around one-third of PLHIV residing in rural sub-Saharan Africa [4-7]. Non-adherence to ART in sub-Saharan Africa is associated with patient-related risk factors and social determinants, including changes in daily activities, forgetting to take ART, lack of health literacy, unwillingness to take ART, unemployment, poverty, HIV-status disclosure concern, HIV-related stigma, lack of clinician trust, poor coping mechanisms and mental health problems [8-11] The effect of these risk factors may be more profound in rural populations, as studies on barriers to care found that PLHIV in rural areas report more and more severe barriers to care than those living in urban areas [12, 13]. This may be of particular relevance to male PLHIV in sub-Saharan African settings, who are at increased risk of various adverse outcomes of treatment, in part stemming from lack of access to care [14–16].

Suboptimal ART adherence can result in virological failure, which has adverse consequences for both individual and public health. Firstly, virological failure of ART is associated with an increased risk of disease progression and reduced survival [17, 18]. Secondly, virological failure is often accompanied by the development of HIV drug resistance [19, 20]. Drug resistance requires switching to more complex and expensive ART regimens, which have a higher pill burden, and often have less tolerable side effects [21, 22]. Finally, virological failure greatly increases the risk of onward transmission of HIV [23].

As roll-out of ART continues to expand in South Africa, efforts are being made to optimize adherence to treatment and treatment efficacy. The recent adoption of dolutegravir (DTG) for all adult PLHIV is expected to improve adherence and efficacy [24]. Effective ART adherence support requires an understanding of the multi-level factors affecting overall adherence, which include socio-demographic, socio-economic, psycho-social and environmental conditions [25]. Knowledge of these factors may be used to identify individuals at risk of non-adherence and to identify factors that can promote ART adherence and improve patient care and programmatic outcomes. The aim of this study is to assess risk factors for adverse outcomes of ART, namely non-adherence and virological failure in rural South African PLHIV. This study also aims to describe the differential risk profiles associated with each of these outcomes.

## Methods

### Design and Procedures

We conducted a prospective cohort study as a sub-study of the Intensified Treatment Monitoring Strategy to Prevent Accumulation of Drug Resistance (ITREMA; Clinicaltrials.gov NCT03357588). ITREMA was an open-label randomized clinical trial evaluating different treatment monitoring strategies for first-line ART, which ran from June 2015 to January 2019 [26]. In this trial, patients initiating ART were to be randomized after 6 months of ART and patients already on ART were randomized at 6 months after the last viral load measurement [26]. The trial protocol can be found in the Supplementary materials (Supplementary material 5).

### Parent Study Control Arm

Patients randomly assigned to this arm were monitored in full concordance with current South African NDoH guidelines in use at the study site. Viral load measurements were performed at month 12 and 24 after start of ART (for newly initiated patients) or at month 12 and 24 after the last viral load measurement (patients already on ART). If a viral load > 1000 copies/ml is detected, the patient was called back for counseling for therapy adherence and repeat viral load measurement, 2 months after the initial viral load measurement. If the repeat viral load measurement was > 1000 copies/ml after adherence counseling, this was taken to be indicative of therapy failure due to development of drug resistance and a switch to second line therapy was made, together with intensified adherence counseling, without verifying the cause of virological failure by performing drug level testing or drug resistance testing. If viral load dropped to < 1000 copies/ml after adherence counseling, the first line treatment was maintained.

### Parent Study Intervention Arm

Patients randomly assigned to this arm were monitored using the investigational intensified monitoring strategy. This strategy consisted of 3-monthly viral load monitoring at month 9, 12, 15, 18, 21 and 24 (after start of ART in initiating patients or after the last viral load measurement in patients on ART). If a viral load measurement > 1000 copies/ml was detected, the patient was called back for a follow-up study visit at the next monthly medication collection visit (4 weeks after detection of elevated viral load). Upon arrival drug level testing was performed, repeated the viral load measurement, and a dried blood spot prepared and stored at room temperature. Procedures following this depended on the result of drug level testing:

If drug levels were detected by drug level testing, the result of the viral load measurement was awaited. If the repeat viral load was > 1000 copies/ml, the dried blood spot was shipped directly by courier to the World Health Organization (WHO) reference laboratory for drug resistance testing. The reference laboratory provided feedback by means of a digital resistance report to the coordinating research physician. The patient would be called back for a second follow-up study visit at the next monthly medication visit (8 weeks after detection of elevated viral load), either for prescription of second-line therapy or continuation of first-line therapy, guided by the result of resistance testing.

If drug level monitoring at the first follow-up visit indicated that drug levels were not detected, intensified counseling was performed at the same visit and first-line therapy was maintained, regardless of the result of the repeat viral load measurement. The patient would not be called back and the next viral load would be performed at the next scheduled 3-monthly time point. However, if the viral load result at this visit was again > 1000 copies/ml, drug resistance testing would be performed regardless of the outcome of drug level testing.

The study was conducted at the Ndlovu medical centre in the rural area of Elandsdoorn where adherence among people living with HIV has not been broadly investigated, which serves the larger Moutse area, situated in the Sekhukhune District Municipality in Limpopo province. It is estimated that the area has about 140–150,000 inhabitants of whom only 4% have tertiary education [27, 28]. According to official statistics, almost half of adults are unemployed, and more than two-thirds of families live below the upper-bound poverty line (UBPL) income of 1183 ZAR ($70.90 per month) [1, 28, 29]. At the time of study, The HIV prevalence in the district was 8.1% of the total population [28], and approximately 3600 PLHIV were receiving HIV treatment and care at Ndlovu Medical Centre. The ITREMA study enrolled adult PLHIV and assessed an intensified treatment monitoring strategy in a randomized comparison with a control group receiving standard-of-care HIV treatment in accordance with the South Africa National Department of Health guidelines [5]. Trial participants were followed up for 96 weeks.

### Sample, Inclusion and Exclusion Criteria

All records of participants who consented to enrolment in the ITREMA trial were included in our sub-study. The trial included adult participants (18 years and older) who were HIV positive and were either ART-naïve and ready to start treatment or had been on treatment for more than 1 year. As the trial intervention was deemed not to interfere with the predictor-outcome relationships assessed in the current study, participants were included regardless of randomization status.

### Data Collection and Measures

Data were collected by a trained research assistant, and included sociodemographic and psychosocial characteristics, self-reported adherence difficulties, pill count and viral load information.

### Sociodemographic Characteristics

Collected information included age, gender, education level, employment status, income, income compositions and household income, number of household members and household composition, and partnership status. Measures were adapted from the South African National Income Dynamics Study and the National Health Nutrition Survey [30, 31]. Income data was entered in South African Rand (ZAR) and was converted to United States Dollar (USD) amounts using the approximate exchange rate at the time of start of study (12.30 ZAR to USD exchange rate, June 2015). Questions regarding food insecurity related to the availability of food in the participant’s household. These included (1) Did your household run out of money to buy food during the past 12 months? (yes/no/do not know), (2) Has it happened in the past 30 days? (yes/no/do not know), (3) Has it happened 5 or more days in the past 30 days? (yes/no/do not know), (4) In the past 12 months, were there times when members of your household went hungry because there was not enough food in the house to eat? (yes/no/do not know), (5) Which were the months (in the last 12 months) in which you experienced a lack of food or money such that one or more members of your household had to go hungry? (January** → **December, do not know). Individuals were labeled to be food insecure if they answer “Yes” to all affirmative household food access scale of occurrence questions.

### Psychosocial Variables

Questionnaire item scores were each evaluated for consistency and distribution (Supplementary material 1). Some composite scores were dichotomized based on skewness of the distribution of the response. Sensitivity analyses of the univariate analyses and multivariable models for each outcome were performed in which all item scores were entered as continuous variables.

Adherence efficacy describes the attitude towards and expectations of the effect of ART that participants had prior to starting treatment. Adherence efficacy was measured with three items from the AIDS Clinical Trials Group (ACTG) questionnaire [32]: “If you do not take this medication exactly as instructed, the HIV in your body will become resistant to HIV medication”, “The medication will have a positive effect on your health”, and “You will be able to take all or most of the medication as directed?”. Responses were measured on a four-point scale, from “not at all” (1) to “extremely sure” (4). Item scores were summed; a higher score indicated higher adherence efficacy (maximum score 12). The 3-item scale had good internal consistency (Cronbach’s alpha = 0.87). Scores were dichotomised and values above 8 were classified as high adherence efficacy.

Support from household and non-household members were each assessed with five items from the Netherlands Kinship Panel Study, and included questions on support in making decisions about work/education, social, leisure time activities, and other personal matters [33], for instance: “To what extent do persons in your household support you?” or “To what extent do family members who do not live in your household support you?” Responses were given on a four-point scale, from ‘no support (1)’ to ‘a lot of support (4)’. The two subscales each had good internal consistency (household members: Cronbach’s alpha = 0.92; non-household members: Cronbach’s alpha = 0.96). Item scores were averaged, and a higher score indicated more support from household or non-household members (maximum score 20). Scores were dichotomised and values above 14 were classified as high household support.

Health literacy was assessed using the Brief Estimate of Health Knowledge and Action (BEHKA) HIV version [34]. This instrument was designed to assess HIV-related health knowledge and the ability to act in accordance with this knowledge, encompassing two subscales: theoretical knowledge (2 items) and operational knowledge (5 items). The following theoretical knowledge items are included: “Is the goal of ARV’s to make the CD4-count go UP or DOWN?”, and “Is the goal of ARV’s to make the viral load go UP or DOWN?”. The theoretical knowledge items were scored as correct or incorrect and correct responses were summed. Operational knowledge questions included: “I don’t take my ARV’s when they make me feel bad”, “I don’t take my ARV’s when I am too tired”, “I don’t take my ARV’s when I am feeling down or low”, “I don’t take my ARV’s because it tastes bad”, and “I don’t take my ARV’s when I feel good”. Responses to the operational knowledge items were given on a three-point scale ranging from “agree” (1) to “disagree” (3). The operational knowledge sub-scale had adequate internal consistency (Cronbach’s alpha = 0.72). Three points were allocated per question where the answer was “disagree”, 1 where the answer was “agree” and 2 points where the answer was “unsure”. Final scores on the operational knowledge items were summed as follows: 0–3 = low, 4–5 = marginal and 6–8 = adequate and a higher score indicated higher health literacy. The calculated composite score as per questionnaire instructions [34, 35] (maximum score 8) was dichotomised and values above 6 were classified as high health literacy.

Clinician trust was assessed with the Revised Helping Alliance Questionnaires [36]. This 11-item instrument is designed to assess the relationship between a patient and their clinician and whether the patient trusts the clinician in terms of shared decision-making, discussing personal matters regarding HIV and ART, communication, and respect. In the context of our study, clinician referred to a clinician providing care to a participant at Ndlovu Medical Centre. The following are examples of items: ‘a good relationship has formed with my clinician’ or ‘I feel the clinician understands me.’ Responses were given on a six-point scale ranging from ‘strongly disagree (1)’ to ‘strongly agree (6)’; The scale had a good internal consistency (Cronbach’s alpha = 0.82). Item scores were summed (maximum score 66), and a higher score represented more trust in the clinician. Summary scores were dichotomised and values above 48 were classified as high clinician trust.

The Coping Inventory for Stressful Situations (CISS-21) was included to measure use of task-, emotion-, and avoidance-oriented coping strategies during a stressful situation, which were each measured with seven items [25, 26]. Task-oriented coping refers to direct action to solve a particular problem (e.g., “I focus on the problem and see how I can solve it”), emotion-oriented coping refers to efforts to modify emotional states caused by stress (e.g., “I blame myself for being too emotional in the situation”), and avoidance-oriented coping refers to efforts to minimize distress by avoiding the problem or finding distracting activities (e.g., “I take some time off and get away from the problem”) [37]. Responses to items were given on a 5-point scale ranging from “never” (1) to “always (5)”. The full scale had good overall internal consistency (Cronbach’s alpha = 0.81), and the internal consistency of the subscales was adequate to good (task-oriented coping: Cronbach’s alpha = 0.88; emotion-oriented coping: Cronbach’s alpha = 0.74; avoidance-oriented coping: Cronbach’s alpha = 0.74). Item scores were summed, and a higher score indicated a more frequent use of the specific coping strategy (maximum score per scale 56). Each subscale was entered as a separate continuous covariable to the statistical analysis.

HIV-related stigma was assessed using the 13-item scale developed by Kalichman et al. [38], which focuses on internalized stigma and stigmatizing beliefs. Items include: “people who have AIDS are dirty”, “most people become HIV positive by being weak or foolish”, “would you mind if people knew if your family member has HIV/AIDS”? Responses were given on a 4-point scale ranging from “strongly disagree” (1) to “strongly agree” (4), with higher scores indicating more experienced stigma. The scale had good internal consistency (Cronbach’s alpha = 0.87). A mean composite score was calculated and dichotomized along the median.

The 9-item Patient Health Questionnaire (PHQ) was used to assesses experienced depression [39]. Participants answered the following question for several depression-related symptoms: “Over the last 2 weeks, how often have you been bothered by any of the following problems?” Examples of symptoms include: “Little interest or pleasure in doing things”, “Feeling down, depressed, or hopeless”, “Trouble falling or staying asleep, or sleeping too much”, “Thoughts that you would be better off dead or of hurting yourself in some way”. Responses were given on a 4-point scale ranging from “not at all” (1), “several days” (2), “more than half the days” (3) to “nearly every day” (4). The results of one participant who refused to answer this questionnaire in full were excluded from this part of the analysis. The scale had adequate internal consistency (Cronbach’s alpha = 0.78). Item scores were summed and dichotomized according to questionnaire instructions [35, 39, 40], with low scores (1–9) indicating minor to mild symptoms of depression and scores > 9 indicating moderate or severe depressive symptoms (maximum score 27).

### ART Adherence and Virological Failure

Self-reported adherence difficulty was measured at 3-monthly intervals between week 12 and 96 of follow-up using three items from the ACTG questionnaire [32] adopted in the CASE self-reported adherence index [41]: “How often do you have difficulty in taking your medication on time”?, with responses given on 4-point scale (1 = all the time, 4 = never, “On average how many days per week would you say that you missed at least one dose of your medication?”, with responses given on a 6-point scale (1 = every day, 6 = never), and “When was the last time you missed taking any of your medications?”, with responses also given on a 6-point scale (1 = past week, 6 = never). Responding ‘never’ to all three questions at all timepoints was taken to indicate no self-reported adherence difficulties. Sensitivity analyses were conducted using the single item “On average how many days per week would you say that you missed at least one dose of your medication?” to assess whether adoption of a more immediate measure of short-term non-adherence would yield different results.

Suboptimal adherence as measured using pill count was measured at 3-monthly intervals between week 12 and 96 of follow-up. Patients were instructed to return with leftover ART tablets. Tablets were counted and the number of doses taken during the last month was calculated as a percentage of the number of doses prescribed by a lay adherence, with 100% indicating complete adherence. Non-adherence was defined as a pill count < 95% in accordance with the threshold used by the WHO [42].

The HIV-RNA load was measured at 6 months (24 weeks), 1 year, and 2 years after initiation of ART in newly initiated participants, and annually in participants already on ART. Virological failure was defined as viremia ≥ 1000 copies/ml, as defined by the WHO [43, 44].

For each outcome, if the outcome definition occurred on at least one timepoint, the patient was marked as having met the outcome definition. Treatment arm allocation in the parent trial was not included as a covariate in the current analysis, as this variable was shown not to be significantly associated with virological failure, self-reported adherence difficulties or suboptimal adherence as measured using pill count [45].

### Data Analysis

Data were analyzed using STATA version 15.1. Frequencies were calculated to describe categorical variables while median and interquartile range were calculated for continuous variables. For univariable analysis of associations between outcomes (self-reported adherence difficulties, suboptimal adherence indicated by pill count and virological failure) and covariables, the Pearson Chi-square or Fischer’s Exact test were used in case of dichotomous and categorical variables, and the Student’s *t*-test in case of continuous covariables. Variables that were associated with the outcome with a significance level of < 0.1 were subsequently included in a multivariable logistic regression analysis to assess independent associations; p < 0.05 was considered statistically significant. Stratified analyses of female and male participants were performed. Adjusted odds ratios (aOR) and their corresponding 95% confidence intervals (95%CI) were reported.

## Results

### Patient Characteristics

Participants’ socio-demographic characteristics are presented in Table 1. Of the 501 participants included, 29.9% were male (150/501). Participants median age was 42 years (IQR 36–49 years); over half the participants were aged 35–49 years (51.3%, 257/501). Over half of participants (58.7%, 294/501) were in a relationship, which included being married, cohabiting, or having a partner but not living together. The majority (81.4%, 408/501) had a secondary (grade 8–12) or tertiary level of education, and 18.6% (93/501) had a primary education level (grade 0–7). More than half of participants (51.1%, 256/501) were unemployed. Over two-thirds of the participant households (69.1%, 346/501) earned less than the current South African minimum wage of 3500.00 ZAR (285 USD) per month, while 6.2% (31/501) households earned above 10,000 ZAR (813 USD) per month. In terms of social grants, about half of the study participants did not receive any grant (51.3%, 257/501), while 22.6% (113/501) received a child support grant, 18.0% (90/501) received an old age grant, and 3.2% (16/501) received a disability grant. The median number of people in participants’ households was 5 (IQR = 3–7). Less than 10% of the study participants reported food insecurity in the last 30 days (8.2%, 41/501). The majority of the study participants had adequate self-efficacy (94.2%, 472/502), high health literacy (98.8%, 487/501), high clinician trust (99.6%, 497/501), good household (86.8%, 434/501) and non-household support (60.9%, 305/501) (Table 1).

**Table 1:** Sample characteristics and univariable analyses of sociodemographic and psychosocial factors associated with self-reported ART adherence difficulties, suboptimal adherence as indicated by pill count < 95% and virological failure among participants in the ITREMA Trial

	Overall sample, n (%) or median [IQR]	Self-reported adherence difficulties (n = 458)					
		Poor self-reported adherence (n = 243, 53.1%)	%	Good self-reported adherence (n = 215, 46.9%)	%	p-value	Chi-square value/t value
Sociodemographic characteristics							
Gender (male)	150 (29.9%)	84	34.6	50	23.3	0.008	7.052
Age (median)	42.0 years [36.0–49.0 years]	42 (37–49)	–	43 (36–50)	–	0.521	0.640
Age (category)							
< 35 years	114 (22.8)	51	21.0	44	20.63		
35–49 years	257 (51.3)	134	55.1	107	50.67		
> 50 years	130 (26.0)	58	23.9	64	28.70		
Relationship status (in a relationship)	294 (58.7)	145	59.7	126	58.6	0.817	0.054
Education (secondary/tertiary)	408 (81.4)	197	81.1	176	81.9	0.828	0.047
Employment (unemployed)	256 (51.1)	124	51.0	108	50.2	0.865	0.029
Household income median (ZAR) (median)	R1600.00 [R700.00–R4200.00]	1500 (660–4000)		1600 (720–4300)		0.851	0.188
Household income per month (category)							
< 3500 ZAR	346 (69.1)	172	70.8	147	68.4		
3500–10,000 ZAR	124 (24.8)	55	22.6	58	27.0		
> 10,000 ZAR	31 (6.2)	16	6.6	10	4.7		
Social grants							
No grants	257 (51.3)	124	51.0	113	52.6	0.925	0.928
Child related grant	113 (22.6)	57	23.5	45	20.9		
Old-age related grant	90 (18.0)	42	17.3	42	19.5		
Other grants	25 (5.0)	12	4.9	9	4.2		
Disability grant	16 (3.2)	8	3.3	6	2.8		
Number of people living together (median)	5 people [3–7 people]	5 (3–7)		5 (3–7)		0.157	1.416
Number of people living together (category)							
1–2	58 (11.6)	29	11.9	22	10.2		
3–5	233 (46.5)	115	47.3	98	45.6		
Above 5	210 (41.9)	99	40.7	95	44.2		
Food insecurity (in the last 30 days)	41 (8.2)	18	7.4	18	8.4	0.731	0.147
Psychosocial characteristics							
Adherence self-efficacy (adequate)	472 (94.2)	226	93.0	208	96.7	0.093	3.213
Health literacy (high)	487 (98.8)	236	98.3	210	99.1	0.508	0.450
Clinician trust (high)	497 (99.6)	242	100.0	213	99.5	0.469	1.133
Household support (good)	434 (86.8)	226	93.0	197	92.1	0.724	0.279
Non-household family support (good)	305 (60.9)	176	72.7	157	73.0	1.000	0.0093
Coping strategy scores							
Task-oriented coping (median)	26 (21–33)	26 (20–32)		27 (21–33)		0.058	1.901
Emotion oriented coping (median)	18 (14–22)	17 (14–21)		18 (15–22)		0.099	1.654
Avoidance oriented coping (median)	15 (12–20)	15.5 (12–20)		15 (12–20)		0.979	− 0.0262
HIV-related (internalized) stigma (stigma)	258 (51.8)	132	54.6	100	46.7	0.096	2.776
Mental health (moderate or severe depressive symptoms)	31 (6.2)	18	7.4	9	4.2	0.146	2.166

	Pill count (n = 458)						Viral load (n = 436)					
	Pill count < 95% (n = 162, 35.4%)	%	Pill count ≥ 95% (n = 296, 64.6%)	%	p-value	Chi-square value/t value	VL ≥ 1000 (n = 68, 15.6%)	%	VL < 1000 (n = 368, 84.4%)	%	p value	Chi-square value/t value
Sociodemographic characteristics												
Gender (male)	58	35.8	76	25.7	0.023	5.187	29	42.7	101	27.5	0.014	6.338
Age (median)	42 (36–48)	–	43 (36–50)		0.250	1.153	41 (36–49)	–	43 (37–50)	–	0.188	1.319
Age (category)												
< 35 years	33	20.4	62	21.0			13	19.1	74	20.1		
35–49 years	95	58.6	146	49.3			38	55.9	193	52.5		
> 50 years	34	21.0	88	29.7			17	25.0	101	27.5		
Relationship status (in a relationship)	97	59.9	174	58.8	0.820	0.052	37	54.4	221	60.1	0.421	0.756
Education (secondary/tertiary)	134	82.7	239	80.7	0.604	0.270	59	86.7	295	80.2	0.201	1.638
Employment (unemployed)	87	53.7	145	49.0	0.334	0.932	40	58.8	182	49.5	0.187	2.015
Household income median (ZAR) (median)	1500 (660–3500)		1800 (710–4500)		0.021	2.378	1500 (510–4500)		1500 (700–4000)		0.629	0.482
Household income per month (category)												
< 3500 ZAR	121	74.7	198	66.9			46	67.7	260	70.7		
3500–10,000 ZAR	36	22.2	77	26.0			18	26.5	88	23.9		
> 10,000 ZAR	5	3.1	21	7.1			4	5.9	20	5.4		
Social grants												
No grants	90	55.7	147	49.7	0.579	3.118	32	47.1	192	52.2	0.338	3.980
Child related grant	32	19.8	70	23.7			18	26.5	81	22.0		
Old-age related grant	31	19.1	53	17.9			10	14.7	72	19.6		
Other grants	6	3.7	15	5.1			5	7.4	14	3.8		
Disability grant	3	1.9	11	3.7			3	4.4	9	2.5		
Number of people living together (median)	5 (3–7)		5 (3–7)		0.053	1.940	5 (4–7)		5 (3–7)		0.295	− 1.049
Number of people living together (category)												
1–2	23	14.2	28	9.5			6	8.8	42	11.4		
3–5	74	45.7	139	47.0			31	45.6	172	46.7		
Above 5	65	40.1	129	43.6			31	45.6	154	41.9		
Food insecurity (in the last 30 days)	16	9.9	20	6.8	0.276	1.407	9	13.2	26	7.1	0.092	2.960
Psychosocial characteristics												
Adherence self-efficacy (adequate)	150	92.6	284	96.0	0.130	2.371	66	97.1	350	95.1	0.752	0.499
Health literacy (high)	155	97.5	291	99.3	0.129	2.644	66	98.5	358	98.6	0.941	0.0054
Clinician trust (high)	161	100.0	294	99.7	1.000	0.547	68	100.0	365	99.7	1.000	0.186
Household support (good)	154	95.1	269	90.9	0.091	1.440	58	85.3	317	86.4	0.630	0.057
Non-household family support (good)	119	73.5	214	72.5	0.912	0.285	41	60.3	227	61.7	0.555	0.047
Coping strategy scores												
Task-oriented coping (median)	25 (20–32)		27 (22–33)		0.025	2.246	25 (20–31)		27 (21–33)		0.051	1.956
Emotion oriented coping (median)	17 (14–21)		18 (14–22)		0.514	0.653	17.5 (14–20)		18 (14–22)		0.059	1.891
Avoidance oriented coping (median)	15 (11–20)		15 (12–20)		0.852	0.187	14 (11–19.5)		15 (12–20)		0.256	1.138
HIV-related (internalized) stigma (stigma)	92	56.8	140	47.6	0.061	3.515	41	60.3	180	49.2	0.094	2.834
Mental health (moderate or severe depressive symptoms)	14	8.7	13	4.4	0.067	3.475	9	13.2	17	4.6	0.009	7.556

Assessments during follow-up of self-reported adherence difficulties and adherence indicated by pill count were available for 458 participants. Viral load data during follow-up were available for 436 participants. Overall, 53.1% (243/458) of participants self-reported adherence difficulties, 35.4% (162/458) had a pill count < 95%, and 15.5% (n = 68/436) experienced virological failure (≥ 1000 copies/ml).

### Overlap Between Study Outcomes

Of all study participants, 7.2% (36/501) met all three study outcomes, 28.3% (142/501) had both self-reported non-adherence and suboptimal pill count, 10.0% (50/501) had both self-reported non-adherence and virological failure, and 7.2% (36/501) reported suboptimal pill count and virological failure.

### Test of Association Analysis

Findings of analyses of covariates of self-reported adherence difficulties, pill count and virological failure are shown in Table 1. Self-reported adherence difficulties were more likely in male compared to female participants (coefficient = 0.56, p = 0.008, chi = 7.052). Suboptimal adherence as indicated by a pill count < 95% was also more likely in male participants (coefficient = 0.48, p = 0.023, chi = 5.187). Suboptimal pill count adherence was inversely associated with household income (coefficient = − 0.063, p = 0.021, chi = 2.378) and use of task-oriented coping (coefficient = − 0.032, p = 0.025, chi = 2.246) and these factors where thus protective against suboptimal pill count adherence. Virological failure was again more likely in male participants (coefficient = 0.68, p = 0.014, chi = 6.338). In addition, there was a strong association between the presence of moderate or severe depressive symptoms and virological failure. These were present in 13.2% [9/68] of participants with virological failure versus 4.6% [17/368] of participants without virological failure (coefficient = 1.14, p = 0.009, chi = 7.556). Univariate findings between the predictor variables and the outcomes demonstrated similar associations (Supplementary material 2) (Tables 2, 3).

**Table 2:** Male: sample characteristics and univariable analyses of sociodemographic and psychosocial factors associated with self-reported ART adherence difficulties, suboptimal adherence as indicated by pill count < 95% and virological failure among participants in the ITREMA Trial

	Overall sample, n (%) or median [IQR]	Self-reported adherence difficulties (n = 458)					
		Poor self-reported adherence (n = 243, 53.1%)	%	Good self-reported adherence (n = 215, 46.9%)	%	p-value	Chi-square value/t value
Sociodemographic characteristics							
Gender (male)	150 (29.9%)	84	34.6	50	23.3	0.008	7.052
Age (median)	43.0 years [37.0–50.0 years]	42 (37.5–50)	–	44 (38–53)	–	0.263	1.124
Age (category)							
< 35 years	28 (18.7)	14	16.7	9	18.0		
35–49 years	77 (51.3)	47	56.0	23	46.0		
> 50 years	45 (30.0)	23	27.3	18	36.0		
Relationship status (in a relationship)	88 (58.7)	53	63.1	29	58.0	0.558	0.342
Education (secondary/tertiary)	108 (80.6)	69	82.1	39	78.0	0.653	0.344
Employment (unemployed)	79 (52.7)	42	50.0	25	50.0	1.000	0.000
Household income median (ZAR) (median)	R1500.00 [R500.00–R3900.00]	1500 (450–3500)		1500 (360–3900)		0.616	− 0.502
Household income per month (category)							
< 3500 ZAR	104 (69.3)	62	73.8	32	64.0		
3500–10,000 ZAR	41 (27.3)	17	20.2	18	36.0		
> 10,000 ZAR	5 (3.3)	5	6.0	0	0.0		
Social grants							
No grants	69 (46.0)	37	44.1	26	52.0	0.719	2.390
Child related grant	35 (23.3)	19	22.6	13	26.0		
Old-age related grant	30 (20.0)	17	20.2	8	16.0		
Other grants	11 (7.3)	7	8.3	2	4.0		
Disability grant	5 (3.3)	4	4.8	1	2.0		
Number of people living together (median)	5 people [3–7 people]	5 (3–6.5)		5 (3–7)		0.385	0.872
Number of people living together (category)							
1–2	21 (14.0)	11	13.1	7	14.0		
3–5	71 (47.3)	42	50.0	23	46.0		
Above 5	58 (38.7)	31	36.9	20	40.0		
Food insecurity (in the last 30 days)	15 (10.0)	9	10.7	2	4.0	0.209	1.875
Psychosocial characteristics							
Adherence self-efficacy (adequate)	139 (92.3)	76	90.5	49	98.0	0.153	2.832
Health literacy (high)	145 (98.0)	80	96.4	50	100.0	0.291	1.849
Clinician trust (high)	150 (100.0)	84	100.0	50	100.0	–	
Household support (good)	122 (81.9)	68	81.0	40	81.6	1.000	0.009
Non-household family support (good)	92 (61.3)	51	60.7	29	58.0	0.856	0.096
Coping strategy scores							
Task-oriented coping (median)	27 (21–34)	26 (20–32)		30.5 (23–34)		0.034	2.144
Emotion oriented coping (median)	18 (14–21)	18 (14–21)		18 (14–21)		0.931	0.086
Avoidance oriented coping (median)	15 (12–20)	16 (12–20)		13 (12–17)		0.095	− 1.684
HIV-related (internalized) stigma (stigma)	86 (57.7)	50	60.2	24	48.0	0.208	1.894
mental Health (moderate or severe depressive symptoms)	11 (7.3)	6	7.1	4	8.0	1.000	0.033

	Pill count (n = 458)						Viral load (n = 436)					
	Pill count < 95% (n = 162, 35.4%)	%	Pill count ≥ 95% (n = 296, 64.6%)	%	p-value	Chi-square value/t value	VL ≥ 1000 (n = 68, 15.6%)	%	VL < 1000 (n = 368, 84.4%)	%	p value	Chi-square value/t value
Sociodemographic characteristics												
Gender (male)	58	35.8	76	25.7	0.023	5.187	29	42.7	101	27.5	0.014	6.338
Age (median)	42 (38–49)	–	44 (37–51.5)		0.364	0.910	40 (36–48)	–	44 (38–51)	–	0.065	1.861
Age (category)												
< 35 years	9	15.5	14	18.4			6	20.7	17	16.8		
35–49 years	34	58.6	36	47.4			16	55.2	50	49.5		
> 50 years	15	25.9	26	34.2			7	24.1	34	33.7		
Relationship status (in a relationship)	37	63.8	45	59.2	0.721	0.291	18	61.4	62	62.1	1.000	0.004
Education (secondary/tertiary)	46	79.3	62	81.6	0.827	0.108	25	86.2	79	78.2	0.436	0.899
Employment (unemployed)	35	60.3	32	42.1	0.055	4.377	19	65.52	45	44.55	0.047	3.961
Household income median (ZAR) (median)	1400 (330–2000)		1850 (650–4585)		0.005	2.860	1500 (0–3500)		1500 (600–3800)		0.692	0.397
Household income per month (category)												
< 3500 ZAR	46	60.5	48	82.8			20	69.0	72	71.3		
3500–10,000 ZAR	26	34.2	9	15.5			7	24.1	26	25.7		
> 10,000 ZAR	4	5.3	1	1.7			2	6.9	3	2.3		
Social grants												
No grants	28	48.3	35	46.1	0.386	4.175	15	51.7	46	45.5	0.770	
Child related grant	10	17.2	22	29.0			8	27.6	24	23.8		
Old-age related grant	12	20.7	13	17.1			3	10.3	21	20.8		
Other grants	6	10.3	3	4.0			2	6.9	6	5.9		
Disability grant	2	3.5	3	4.0			1	3.5	4	4.0		
Number of people living together (median)	4 (3–6)		5 (3–7)		0.245	1.167	5 (4–7)		5 (3–7)		0.746	− 0.324
Number of people living together (category)												
1–2	8	13.8	10	13.1			2	6.9	15	14.9		
3–5	29	50.0	36	47.4			17	58.6	47	46.5		
Above 5	21	36.2	30	39.5			10	34.5	39	38.6		
Food insecurity (in the last 30 days)	9	15.5	2	2.6	0.010	7.248	6	20.7	5	5.0	0.015	7.205
Psychosocial characteristics												
Adherence self-efficacy (adequate)	54	93.1	71	93.4	1.000	0.005	28	96.6	94	93.1	0.683	0.473
Health literacy (high)	55	96.5	75	98.7	0.576	0.711	27	96.4	99	98.0	0.523	0.244
Clinician trust (high)	58	100.0	76	100.0	–		29	100.0	101	100.0	–	
Household support (good)	46	80.70	62	81.58	1.000	0.016	23	79.3	81	81.0	0.796	0.041
Non-household family support (good)	32	55.17	48	63.16	0.378	0.872	17	58.6	61	60.4	1.000	0.030
Coping strategy scores												
Task-oriented coping (median)	26.5 (20–34)		27 (22–34)		0.518	0.647	26 (19–31)		27 (21–34)		0.131	1.521
Emotion oriented coping (median)	18 (14–21)		18 (14–22)		0.743	0.329	18 (14–20)		17 (14–22)		0.549	0.601
Avoidance oriented coping (median)	14.5 (11–20)		15 (12–19)		0.560	0.584	14 (10–17)		15 (13–19)		0.159	1.417
HIV-related (internalized) stigma (stigma)	34	58.6	40	53.3	0.599	0.371	18	62.1	53	53.0	0.406	0.747
Mental Health (moderate or severe depressive symptoms)	6	10.3	4	5.3	0.328	1.230	4	13.8	5	5.0	0.111	2.734

**Table 3:** Female: Sample characteristics and univariable analyses of sociodemographic and psychosocial factors associated with self-reported ART adherence difficulties, suboptimal adherence as indicated by pill count < 95% and virological failure among participants in the ITREMA Trial

	Overall sample, n (%) or median [IQR]	Self-reported adherence difficulties (n = 458)					
		Poor self-reported adherence (n = 243, 53.1%)	%	Good self-reported adherence (n = 215, 46.9%)	%	p-value	Chi-square value/t value
Sociodemographic characteristics							
Gender (female)	351 (70.1%)	159	65.4	165	76.7	0.008	7.052
Age (median)	42.0 years [35.0–49.0 years]	42 (36–49)	–	43 (35–50)	–	0.829	0.216
Age (category)							
< 35 years	86 (24.5)	37	23.3	35	21.2		
35–49 years	180 (51.3)	87	54.7	84	50.9		
> 50 years	85 (24.2)	35	22.0	46	27.9		
Relationship status (in a relationship)	206 (58.7)	92	57.9	97	58.8	0.910	0.029
Education (secondary/tertiary)	265 (81.8)	128	80.5	137	83.0	0.568	0.347
Employment (unemployed)	177 (50.4)	82	51.6	83	50.3	0.825	0.052
Household income median (ZAR) (median)	R1800.00 [R1000.00–R4345.00]	1600 (720–4000)		1900 (1050–4345)		0.759	0.308
Household income per month (category)							
< 3500 ZAR	242 (69.0)	110	69.1	115	69.7		
3500–10,000 ZAR	83 (23.7)	38	23.9	40	24.2		
> 10,000 ZAR	26 (7.4)	11	7.0	10	6.1		
Social grants							
No grants	188 (53.6)	87	54.7	87	52.7	0.695	2.221
Child related grant	78 (22.2)	38	23.9	32	19.4		
Old-age related grant	60 (17.1)	25	15.7	34	20.6		
Other grants	14 (4.0)	5	3.1	7	4.2		
Disability grant	11 (3.1)	4	2.5	5	3.0		
Number of people living together (median)	5 people [3–7 people]	5 (3–7)		5 (3–7)		0.345	0.945
Number of people living together (category)							
1–2	37 (10.5)	18	11.3	15	9.0		
3–5	162 (46.2)	73	45.9	75	45.5		
Above 5	152 (43.3)	68	42.8	75	45.5		
Food insecurity (in the last 30 days)	26 (7.4)	9	5.7	16	9.7	0.213	1.852
Psychosocial characteristics							
Adherence self-efficacy (adequate)	333 (94.9)	150	94.3	159	96.4	0.437	0.751
Health literacy (high)	342 (99.1)	156	99.4	160	98.8	1.000	0.306
Clinician trust (high)	347 (99.4)	158	100.0	163	99.4	1.000	0.966
Household support (good)	312 (88.9)	68	81.0	40	81.6	1.000	1.293
Non-household family support (good)	213 (60.7)	97	61.0	101	61.2	1.000	0.001
Coping strategy scores							
Task-oriented coping (median)	26 (21–32)	25.5 (20.5–32)		27 (21–33)		0.339	0.958
Emotion oriented coping (median)	18 (14–22)	17 (14–21)		19 (15–22)		0.078	1.767
Avoidance oriented coping (median)	15 (12–20)	15 (11–19)		16 (12–20)		0.367	0.903
HIV-related (internalized) stigma	172 (49.3)	82	51.6	76	46.3	0.374	0.884
Mental Health (moderate or severe depressive symptoms)	20 (5.7)	12	7.6	5	3.0	0.082	3.373

	Pill count (n = 458)						Viral load (n = 436)					
	Pill count < 95% (n = 162, 35.4%)	%	Pill count ≥ 95% (n = 296, 64.6%)	%	p-value	Chi-square value/t value	VL ≥ 1000 (n = 68, 15.6%)	%	VL < 1000 (n = 368, 84.4%)	%	p value	Chi-square value/t value
Sociodemographic characteristics												
Gender (female)	104	64.2	220	74.3	0.023	5.187	39	57.4	267	72.6	0.014	6.338
Age (median)	42.5 (35–48)	–	42.5 (36–50)		0.362	0.913	42 (36–49)	–	43 (36–49)	–	0.746	0.324
Age (category)												
< 35 years	24	23.1	48	21.8			7	18.0	57	21.4		
35–49 years	61	58.7	110	50.0			22	56.4	143	53.5		
> 50 years	19	18.2	62	28.2			10	25.6	67	25.1		
Relationship status (in a relationship)	60	57.7	129	58.6	0.904	0.026	19	48.7	159	59.6	0.226	1.641
Education (secondary/tertiary)	88	84.6	177	80.5	0.441	0.821	34	87.2	216	80.9	0.505	0.898
Employment (unemployed)	52	50.0	113	51.4	0.905	0.053	21	53.9	137	51.3	0.767	0.088
Household income median (ZAR) (median)	1650 (1000–4000)		1800 (1000–4422)		0.375	0.889	1700 (1000–5200)		1600 (720–4200)		0.899	0.127
Household income per month (category)												
< 3500 ZAR	73	70.1	152	69.1			26	66.7	188	70.4		
3500–10,000 ZAR	27	26.0	51	23.2			11	28.2	62	23.2		
> 10,000 ZAR	4	3.9	17	7.7			2	5.1	17	6.4		
Social grants												
No grants	62	59.6	112	50.9	0.053	8.503	17	43.6	146	54.7	0.214	4.760
Child related grant	22	21.2	48	21.8			10	25.6	57	21.4		
Old-age related grant	19	18.3	40	18.2			7	18.0	51	19.1		
Other grants	0	0	12	5.5			3	7.7	8	3.0		
Disability grant	1	1.0	8	3.6			2	5.1	5	1.9		
Number of people living together (median)	5 (3–7)		5 (3–7)		0.160	1.407	6 (4–7)		5 (3–7)		0.203	− 1.276
Number of people living together (category)												
1–2	15	14.4	18	8.18			4	10.3	27	10.1		
3–5	45	43.3	103	46.8			14	35.9	125	46.8		
Above 5	44	42.3	99	45.0			21	53.9	115	43.1		
Food insecurity (in the last 30 days)	7	6.7	18	8.2	0.824	0.209	3	7.7	21	7.9	1.000	0.0014
Psychosocial characteristics												
Adherence self-efficacy (adequate)	96	92.3	213	96.8	0.090	3.254	38	97.4	256	95.9	1.000	0.219
Health literacy (high)	100	98.0	216	99.5	0.241	1.676	39	100.0	259	98.9	1.000	0.451
Clinician trust (high)	103	100.0	218	99.5	1.000	0.472	39	100.0	264	99.6	1.000	0.148
Household support (good)	98	94.2	191	86.8	0.055	4.027	35	89.7	236	88.4	1.000	0.062
Non-household family support (good)	69	66.4	129	58.6	0.222	1.766	24	61.5	166	62.2	1.000	0.006
Coping strategy scores												
Task-oriented coping (median)	25 (20–31)		27 (22–33)		0.017	2.391	24 (20–31)		27 (21–33)		0.193	1.306
Emotion oriented coping (median)	17 (14–21)		18 (14–22)		0.657	0.444	17 (14–19)		18 (14–22)		0.068	1.831
Avoidance oriented coping (median)	15 (11–20)		15 (12–20)		0.833	− 0.210	14 (11–21)		16 (12–20)		0.789	0.268
HIV-related (internalized) stigma	58	55.8	100	45.7	0.096	2.883	23	59.0	172	47.7	0.231	1.716
Mental health (moderate or severe depressive symptoms)	8	7.8	9	4.1	0.186	1.901	5	12.8	12	4.5	0.051	4.462

### Multivariable Analysis

Results of multivariable logistic regression analyses of correlates of self-reported adherence difficulties, suboptimal adherence indicated by pill count and virological failure are shown in Table 4. For self-reported adherence difficulties, the only independent risk factor was male gender (aOR 1.78 [95%CI 1.17–2.71]; p = 0.007, z = 2.67). Male gender also was an independent risk factor for poor adherence as indicated by pill count < 95% (aOR 1.57 [95%CI 1.02–2.41]; p = 0.040, z = 2.06), while higher household income (aOR 0.94 [95%CI 0.89–0.99]; p = 0.030, z = − 2.26) and higher task-oriented coping (aOR 0.97 [95%CI 0.94–1.00]; p = 0.031, z = − 2.16) were found to be protective factors against suboptimal adherence indicated by pill count < 95%. Independent risk factors for virological failure were male gender (aOR 1.95 [95%CI 1.13–3.36]; p = 0.017, z = 2.39) and moderate or severe depressive symptoms (aOR 2.92 [95%CI 1.17–7.79]; p = 0.021, z = 2.30). Stratified analyses by sex showed that virological failure was more likely in males who reported food insecurity in the last 30 days (aOR 5.74 [95%CI 1.49–22.05]; p = 0.011, z = 2.54) and in females reporting depression (aOR 3.32 [95%CI 1.02–10.82]; p = 0.046, z = 1.99) (Tables 5, 6).

**Table 4:** Multivariable analyses of sociodemographic and psychosocial factors associated with of self-reported ART adherence difficulties, suboptimal adherence as indicated by pill count < 95% and virological failure among participants in the ITREMA Trial

Variable	Self-reported adherence difficulties			Pill count < 95%			Virological failure (≥ 1000 copies/ml)		
	Adjusted odds ratio (95% CI)	p-value	z value	Adjusted odds ratio (95% CI)	p-value	z value	Adjusted odds ratio (95% CI)	p-value	z value
Gender									
Female	Ref	–	–	Ref	–		Ref	–	
Male	1.78 (1.17–2.71)	0.007	2.67	1.57 (1.02–2.41)	0.040	2.06	1.95 (1.13–3.36)	0.017	2.39
Household income (per 1000 ZAR)	–	–	–	0.94 (0.89–0.99)	0.024	− 2.26	–	–	
Number of people living together	–	–	–	0.94 (0.87–1.01)	0.074	− 1.78	–	–	–
Adherence self-efficacy									
Inadequate adherence self-efficacy	Ref		–	–	–	–	–	–	–
Adequate adherence self-efficacy	0.56 (0.22–1.44)	0.231	− 1.20	–	–	–	–	–	–
Household family support									
Poor household family support	–	–	–	Ref	–	–	–	–	–
Household family support	–	–	–	1.54 (0.82–2.88)	0.179	1.40	–	–	–
Coping strategy scores (CISS)									
Task-oriented coping	0.80 (0.54–1.19)	0.248	− 1.16	0.97 (0.94–1.00)	0.031	− 2.16	0.98 (0.94–1.01)	0.230	− 1.20
Emotion-oriented coping	0.77 (0.53–1.13)	0.234	− 1.19	–	–	–	0.95 (0.90–1.01)	0.093	− 1.68
Food insecurity in the last 30 days									
No reported food insecurity in the last 30 days	–	–	–	–	–	–	Ref	–	
Reported food insecurity in the last 30 days	–	–	–	–	–	–	1.76 (0.75–4.10)	0.193	1.30
HIV related stigma									
No reported stigma	Ref	–	–	Ref		–	Ref	–	–
Reported stigma	1.18 (0.80–1.75)	0.401	0.84	1.22 (0.81–1.85)	0.341	0.52	1.29 (0.64–2.61)	0.471	0.72
Mental health									
Minimal or no depressive symptoms	–	–	–	Ref	–	–	Ref	–	–
Moderate or severe depressive symptoms	–	–	–	1.93 (0.85–4.37)	0.114	1.62	2.92 (1.17–7.29)	0.021	2.30

**Table 5:** Male: multivariable analyses of sociodemographic and psychosocial factors associated with of self-reported ART adherence difficulties, suboptimal adherence as indicated by pill count < 95% and virological failure among participants in the ITREMA Trial

Variable	Self-reported adherence difficulties			Pill count < 95%			Virological failure (≥ 1000 copies/ml)		
	Adjusted odds ratio (95% CI)	p-value	z value	Adjusted odds ratio (95% CI)	p-value	z value	Adjusted odds ratio (95% CI)	p-value	z value
Age	–	–		–	–	–	0.97 (0.93–1.01)	0.123	− 1.54
Employment (unemployed)	–	–		1.24 (0.56–2.74)	0.592	0.54	1.86 (0.76–4.58)	0.177	1.35
Household income (per 1000 ZAR)	–	–	–	0.84 (0.72–0.98)	0.028	− 2.20	–	–	–
Coping strategy scores (CISS)									
Task-oriented coping	0.95 (0.90–1.00)	0.041	− 2.04	–	–		–	–	–
Emotion-oriented coping	1.00 (0.93–1.08)	0.929	0.09	–	–		–	–	–
Food insecurity in the last 30 days									
No reported food insecurity in the last 30 days	–	–		–	–	–	Ref	–	–
Reported food insecurity in the last 30 days	–	–	–	6.34 (1.18–34.09)	0.031	2.15	3.98 (1.07–14.89)	0.040	2.05

**Table 6:** Female: multivariable analyses of sociodemographic and psychosocial factors associated with of self-reported ART adherence difficulties, suboptimal adherence as indicated by pill count < 95% and virological failure among participants in the ITREMA Trial

Variable	Self-reported adherence difficulties			Pill count < 95%			Virological failure (≥ 1000 copies/ml)		
	Adjusted odds ratio (95% CI)	p-value	z value	Adjusted odds ratio (95% CI)	p-value	z value	Adjusted odds ratio (95% CI)	p-value	z value
Social grants									
Child related grant	–	–	–	Ref	–		–	–	–
Disability grant	–	–	–	0.21 (0 .02–1.96)	0.173	− 1.36	–	–	–
No grants	–	–	–	1.37 (0 .73–2.56)	0.323	0.99	–	–	–
Old-age related grant	–	–	–	1.46 (0 .65–3.29)	0.356	0.92	–	–	–
Other grants	–	–	–	1	–	–	–	–	–
Adherence self-efficacy									
Inadequate adherence self-efficacy	–	–	–	Ref	–	–	–	–	–
Adequate adherence self-efficacy	–	–	–	0.36 (0.11–1.21)	0.100	− 1.64	–	–	–
Household family support									
Poor household family support	–	–	–	Ref	–	–	–	–	–
Household family support	–	–	–	2.43 (0.95–6.20)	0.064	1.85	–	–	–
Coping strategy scores (CISS)									
Task-oriented coping	–	–	–	0.96 (0.92–1.00)	0.045	− 2.01	–	–	
Emotion-oriented coping	0.96 (0.92–1.00)	0.056	− 1.91	–	–	–	0.93 (0.87–1.00)	0.046	− 1.99
HIV related stigma									
No reported stigma	–	–	–	Ref	–	–	–	–	–
Reported stigma	–	–	–	1.13 (0.66–1.92)	0.653	0.45	–	–	–
Mental health									
Minimal or no depressive symptoms	Ref	–	–	–	–	–	Ref	–	–
Moderate or severe depressive symptoms	2.93 (0.99–8.64)	0.051	1.95	–	–	–	3.58 (1.15–11.10)	0.027	2.21

### Sensitivity Analyses

When all psychosocial characteristics were entered as continuous measures to univariate and multivariable analysis, results for the outcomes of self-reported adherence difficulties and virological failure remained essentially unchanged (Supplementary materials 2, 3). For suboptimal adherence as indicated by pill count < 95%, the associations in univariate and multivariable analysis with gender and household income remained consistent. However, the association with task-oriented coping remained present in univariate analysis but not in multivariable analysis. Instead, the multivariable analysis for this outcome revealed associations with health literacy and household family support (Supplementary materials 2, 3).

Sensitivity analysis using a self-reported adherence variable that considers only short-term adherence demonstrated a prevalence of self-reported short-term non-adherence of 51.3% (235/458) versus a prevalence of 53.1% (243/458) for the definition of self-reported adherence difficulties that was used in the main analysis. Univariate associations and multivariable model results with this outcome were consistent with the results of the main analysis (Supplementary materials 4).

## Discussion

Our study has assessed rates and sociodemographic and psychosocial factors associated with non-adherence as measured through self-report and pill count and virological failure among PLHIV in a South African rural population. We identified several demographic, socio-economic, and behavioural risk factors for non-adherence and virological failure and showed that there is limited overlap of markers of adherence with virological failure. We found that male gender was an independent risk factor for all outcomes. Depressive symptoms were independently associated with virological failure while household income and task-oriented coping score were protective against suboptimal pill-count adherence.

In multivariable analyses we found that self-reported adherence difficulties, suboptimal pill count adherence and virological failure were more likely in men than women. The finding of increased problems with adherence to ART in men is consistent with other studies conducted in rural settings and may reflect poorer healthcare behaviour in men [15, 46-53]. Several studies performed in different cultural contexts have identified underlying reasons for the generally poorer healthcare behavior of men [4, 54–56]. For men in rural settings in particular these include lack of time, poor healthcare access due to social constructions of masculinity, underlying cultural reasons, distance needed to travel to access care, and the lack of male care providers [4, 15, 54, 55, 57, 58]. Men from rural settings report more severe barriers to care than their urban counterparts [13, 59], highlighting the need to acknowledge how masculinity serve as a barrier for rural men’s access to ART services and prioritize rural men for interventions promoting ART adherence [55].

Suboptimal adherence as indicated by pill count was associated with low household income, as also found by other studies [41, 60]. These findings confirm that despite the scale up of free ART in South Africa, financial constraints remain a barrier to ART adherence. Concerns around low household income in rural settings are centered on cost of seeking treatment, the distance needed to travel to access care, reliance on traditional medicine and the cost of food [41]. At first sight, our finding that in stratified analysis the risk of virological failure was higher among male participants with food insecurity seems to be a result of this same dynamic. However, this finding is at odds with other studies reporting that women, not men, are less favored in terms of household food distribution and that mechanisms for how food insecurity impacted adherence were generally similar among women and men [61, 62]. Nonetheless, our results suggest that there is a link between gender, food insecurity and ART adherence, heightening the importance of addressing food insecurity as part of comprehensive care among PLHIV. Further research should also examine how the negative impact of food unavailability on adherence in food-stressed households can be mitigated. Programmatic models that have been successful in rural settings, aiming to improve food security and nutrition in an HIV context include: (1) Nutrition supplementation interventions targeted to undernourished PLHIV, often using specialized foods, with nutrition assessment, counselling and support as a central component targeted to all PLHIV regardless of nutrition status. (2) Safety nets (food, cash transfer or vouchers), targeted to HIV-affected households and individuals (such as Orphans and Vulnerable Children) to improve household food security, mitigate the impact of HIV, and (3) Livelihood interventions targeted to PLHIV households or communities heavily affected by the AIDS epidemic [63].

In this cohort, better adherence as indicated by pill count was associated with increased use of task-oriented coping. This was more evident in female participants. This suggests that counselling strategies addressing specific coping styles could have a positive effect on ART treatment outcomes. Moreover, it is likely that there also are indirect associations between coping strategies, sex, and adherence to treatment through markers of mental health. More research is needed to assess these relationships and guide future interventions.

Furthermore, we found that the risk of virological failure was higher among participants with moderate or severe depressive symptoms. In stratified analysis, we found that while the prevalence of depressive symptoms was similar between men and women, the association was significant among female participants only. These findings are comparable to those of several studies assessing risk factors for non-adherence in PLHIV, which also found evidence that depression was associated with poor outcomes for HIV-infected patients on ART, especially in women [64, 65]. Our results suggest that, despite substantial progress made in quality and access to HIV related health care [66] as seen with recent transition to a DTG-based regimen [67], depression and other mental health problems remain underdiagnosed and often untreated among rural PLHIV [13, 68]. There hence remains a critical need to screen for and treat depressive symptoms in PLHIV. The association between depression, sex and markers of suboptimal adherence indicate that screening for mental health problems should be considered as an integral part of adherence counselling, and that treatment of these problems could potentially improve adherence to ART.

We encountered high levels of both self-reported adherence difficulties as well as suboptimal adherence as measured through pill count in this setting. In contrast, rates of virological failure in this cohort were more limited. While self-reported adherence difficulties and suboptimal pill count results were significantly correlated with each other and with virological failure, overlap between these outcomes was limited. Sensitivity analysis showed similar findings for self-reported adherence difficulties. Various studies have reported significant correlations between objective and self-report measures of ART adherence [69-71], even though some studies conducted in developed and developing settings suggest that viral load is more likely to be accurate in terms of reflecting true adherence rates than self-reported adherence [8, 72]. Previous research conducted in sub-Saharan African countries has also found that self-reported adherence tended to over or under-estimate adherence and is not necessarily associated with the virological suppression status of patients [73-76]. Factors that may affect the reliability of self-reported adherence and pill counts are numerous, and include variation in measurement methods and thresholds, social desirability bias, healthcare worker trust, and recall error.

### Limitations and Strengths

This study included participants receiving clinical care in a medical centre in rural Limpopo, South Africa. Therefore, findings from this study may not be generalizable to other rural settings in South Africa, or to other country settings. While the study assessed a broad range of psychosocial factors, the many individual, social and structural factors that may be of influence cannot feasibly be assessed in a single study. Therefore, the scope of covariates, while broad, by definition, and inevitably is limited. Potential covariates of ART adherence were assessed through self-report, which may have been affected by memory bias and social desirability bias. This study also highlights the practical limitations of conducting pill counts. In many cases patients forgot to take their left-over medication to their clinical visit. Previous studies used unannounced pill count to avoid this practical limitation, but this may not be feasible in practice.

## Conclusion

This study in a rural community of people with HIV found that PLHIV who were male, had low household income or experienced moderate or severe depressive symptoms were at increased risk of suboptimal adherence and/or virological failure, and may benefit from additional ART adherence support. Sex-specific risk factors that were identified included depressive symptoms in women and food insecurity in men. Task-oriented coping was protective against suboptimal adherence as indicated by pill count. While the rate of self-reported adherence difficulties was high, there was limited overlap between risk factors for non-adherence and virological failure. The high levels of self-reported adherence difficulties would point to a possible self-reporting bias resulting in an over-estimation of bias adherence problems. Our identification of socio-demographic and psychosocial risk factors can guide the targeting of adherence support interventions to rural populations, as well as highlight the factors underlying adherence problems that should be addressed in such interventions. Our findings contribute to the available knowledge on risk factors for adverse outcomes of ART in rural populations and may contribute to the ongoing development of ‘rural proof’ healthcare policies currently being introduced in South Africa, such as National Health Insurance and the new 2030 Human Resources for Health Strategy.

### Supplementary Information

Below is the link to the electronic supplementary material.

Supplementary file1 (DOCX 80 KB)

Supplementary file2 (DOCX 19 KB)

Supplementary file3 (DOCX 17 KB)

Supplementary file4 (DOCX 25 KB)

Supplementary file5 (PDF 1708 KB)

## Data Availability

The datasets used or analysed for the current study are available from the corresponding author on reasonable request.
