# Supplementary material for: Predictors of Treatment Adherence and Virological Failure Among People Living with HIV Receiving Antiretroviral Therapy in a South African Rural Community: A Sub-study of the ITREMA Randomised Clinical Trial
Source: AIDS Behav. 2023 Jun 29;27(12):3863–85. doi: 10.1007/s10461-023-04103-2 (PMC10598166; doi:10.1007/s10461-023-04103-2)
Supplement: Supplementary file 1 — Supplementary file1 (DOCX 80 KB) [file 10461_2023_4103_MOESM1_ESM.docx]

Supplementary material 1: Histograms, median, mean and Cronbach for psychosocial variables

Supplementary graph 1: Distribution of adherence self-efficacy scores for the ITREMA participants,

*Cronbach’s alpha = 0.87*

Supplementary graph 2: Distribution of household support scores for the ITREMA participants.

*Cronbach’s alpha = 0.92*

Supplementary graph 3: Distribution of non-household support scores for the ITREMA participants.

*Cronbach’s alpha = 0.96*

Supplementary graph 4: Distribution of clinician trust scores for the ITREMA participants

*Cronbach’s alpha = 0.72*

Supplementary graph 5: Distribution of health literacy scores for the ITREMA participants.

*Cronbach’s alpha = 0.82*

Supplementary graph 6: Distribution of task oriented coping scores for the ITREMA participants.

*Cronbach’s alpha = 0.88*

Supplementary graph 7: Distribution of emotion oriented coping scores for the ITREMA participants.

*Cronbach’s alpha = 0.74*

Supplementary graph 8: Distribution of avoidance oriented coping scores for the ITREMA participants.

*Cronbach’s alpha = 0.74*

Supplementary graph 9: Distribution of HIV related stigma scores for the ITREMA participants.

*Cronbach’s alpha = 0.87*

Supplementary graph 10: Distribution of Mental health (depressive symptoms) scores for the ITREMA participants.

*Cronbach’s alpha = 0.78*
