# Supplementary material for: Predictors of Treatment Adherence and Virological Failure Among People Living with HIV Receiving Antiretroviral Therapy in a South African Rural Community: A Sub-study of the ITREMA Randomised Clinical Trial
Source: AIDS Behav. 2023 Jun 29;27(12):3863–85. doi: 10.1007/s10461-023-04103-2 (PMC10598166; doi:10.1007/s10461-023-04103-2)
Supplement: Supplementary file 2 — Supplementary file2 (DOCX 19 KB) [file 10461_2023_4103_MOESM2_ESM.docx]

Supplementary material 2: Univariate analyses of sociodemographic and psychosocial factors associated with self-reported ART adherence difficulties, suboptimal adherence as indicated by pill count <95% and virological failure among participants in the ITREMA Trial

| Sociodemographic characteristics | | Self-reported adherence difficulties | | | Pill count <95% | | | Virological failure (≥1000 copies/ml) | | |
| --- | --- | --- | --- | --- | --- | --- | --- | --- | --- | --- |
|  |  | Unadjusted Odds  Ratio (95% CI) | p-value | z value | Unadjusted Odds  Ratio (95% CI) | p-value | z value | Unadjusted Odds  Ratio (95% CI) | p-value | z value |
| Gender (male) | 150 (29.9%) | 1.74 (1.15-2.63) | 0.008 | 2.64 | 1.61 (1.07-2.44) | 0.023 | 2.27 | 1.97 (1.15-3.35) | 0.013 | 2.49 |
| Age (median) | 42.0 years [36.0-49.0 years] | 0.994 (0.997-1.011) | 0.521 | -0.64 | 0.99 (0.97-1.01) | 0.249 | -1.15 | 0.98 (0.96-1.01) | 0.188 | -1.32 |
| Relationship status (in a relationship) | 294 (58.7) | 1.05 (0.72-1.52) | 0.817 | 0.23 | 1.05 (0.71-1.55) | 0.820 | 0.23 | 0.79 (0.47-1.34) | 0.385 | -0.87 |
| Education (primary vs secondary/tertiary level) | 470 (93.8) | 0.76 (0.36-1.60) | 0.469 | -0.72 | 0.77 (0.34-1.72) | 0.525 | -0.63 | 0.82 (0.28-2.44) | 0.724 | -0.35 |
| Employment  (unemployed) | 256 (51.1) | 1.03 (0.72-1.49) | 0.865 | 0.17 | 1.21 (0.82-1.77) | 0.335 | 0.96 | 1.46 (0.86-2.47) | 0.157 | 1.41 |
| Household income median (ZAR) (median) | R1600.00 [R700.00-R4200.00 | 1.00 (0.95-1.03) | 0.851 | -0.19 | 0.94 (0.89-0.99) | 0.021 | -2.31 | 0.98 (0.92-1.05) | 0.629 | -0.48 |
| Social grants |  | | | | | | | | | |
| Child related grant | 113 (22.6) | 1 | - | - | 1 | - | - | 1 | - | - |
| Disability grant | 16 (3.2) | 1.05 (0.34-3.25) | 0.090 | 0.929 | 0.60 (0.16-2.29) | 0.451 | -0.75 | 1.50 (0.37-6.10) | 0.571 | 0.57 |
| No grants | 257 (51.3) | 0.87 (0.54-1.38) | 0.547 | -0.60 | 1.34 (0.82-2.19) | 0.579 | 1.16 | 0.75 (0.40-1.41) | 0.373 | -0.89 |
| Old-age related grant | 90 (18.0) | 0.79 (0.44-1.41) | 0.424 | -0.80 | 1.28 (0.70-2.35) | 0.428 | 0.79 | 0.63 (0.27-1.44) | 0.270 | -1.10 |
| Other grants | 25 (5.0) | 1.05 (0.41-2.72) | 0.916 | 0.11 | 0.88 (0.31-2.46) | 0.800 | -0.25 | 1.61 (0.51-5.03) | 0.415 | 0.81 |
| Number of people living together (median) | 5 people [3-7 people] | 0.95 (0.89-1.02) | 0.158 | -1.41 | 0.93 (0.87-1.00) | 0.054 | -1.93 | 1.05 (0.96-1.15) | 0.295 | 1.05 |
| Food insecurity (in the last 30 days) | 41 (8.2) | 0.88 (0.44-1.73) | 0.702 | -0.38 | 1.51 (0.76-3.01) | 0.238 | 1.18 | 2.01 (0.90-4.50) | 0.091 | 1.69 |
| Psychosocial characteristics | | | | | | | | | | |
| Adherence self-efficacy  (adequate) | 472 (94.2) | 0.45 (0.18-1.10) | 0.080 | -1.75 | 0.53 (0.23-1.20) | 0.129 | -1.52 | 1.70 (0.38-7.49) | 0.485 | 0.70 |
| Health literacy (high) | 487 (98.8) | 0.56 (0.10-3.10) | 0.508 | -0.66 | 0.27 (0.05-1.47) | 0.129 | -1.52 | 0.92 (0.11-8.02) | 0.941 | -0.07 |
| Clinician trust (high) | 497 (99.6) | 1 | - | - | 1 | - | - | 1 | - | - |
| Household support | 434 (86.8) | 1.16 (0.67-1.99) | 0.597 | 0.53 | 1.44 (0.79-2.62) | 0.232 | 1.20 | 0.91 (0.44-1.91) | 0.812 | -0.24 |
| Non-household family support (good) | 305 (60.9) | 1.02 (0.70-1.48) | 0.923 | 0.10 | 1.11 (0.75-1.65) | 0.593 | 0.53 | 0.94 (0.56-1.60) | 0.829 | -0.22 |
| Coping strategy scores |  | | | | | | | | | |
| Task-oriented coping  (median) | 26 (21-33) | 0.97 (0.95-1.00) | 0.058 | -1.89 | 0.97 (0.94-1.00) | 0.026 | -2.23 | 0.96 (0.93-1.00) | 0.052 | -1.94 |
| Emotion oriented coping  (median) | 18 (14-22) | 0.97 (0.94-1.01) | 0.099 | -1.65 | 0.99 (0.95-1.03) | 0.513 | -0.65 | 0.95 (0.90-1.00) | 0.060 | -1.88 |
| Avoidance oriented coping (median) | 15 (12-20) | 1.00 (0.97-1.03) | 0.979 | 0.03 | 1.00 (0.96-1.03) | 0.851 | -0.19 | 0.97 (0.93-1.02) | 0.256 | -1.14 |
| HIV-related  (internalized) stigma  (stigma) | 258 (51.8) | 1.37 (0.95-1.98) | 0.096 | 1.66 | 1.45 (0.98-2.13) | 0.061 | 1.87 | 1.57 (0.93-2.66) | 0.094 | 1.67 |
| Mental Health (moderate or severe depressive  symptoms) | 31 (6.2) | 1.84 (0.81-4.19) | 0.146 | 1.45 | 2.07 (0.95-4.53) | 0.067 | 1.83 | 3.14 (1.34-7.38) | 0.009 | 2.63 |
