# Supplementary material for: Predictors of Treatment Adherence and Virological Failure Among People Living with HIV Receiving Antiretroviral Therapy in a South African Rural Community: A Sub-study of the ITREMA Randomised Clinical Trial
Source: AIDS Behav. 2023 Jun 29;27(12):3863–85. doi: 10.1007/s10461-023-04103-2 (PMC10598166; doi:10.1007/s10461-023-04103-2)
Supplement: Supplementary file 3 — Supplementary file3 (DOCX 17 KB) [file 10461_2023_4103_MOESM3_ESM.docx]

Supplementary material 3: Sensitivity analysis: Multivariable analyses of sociodemographic and psychosocial factors associated with of self-reported ART adherence difficulties, suboptimal adherence as indicated by pill count <95% and virological failure among participants in the ITREMA Trial

| **Variable** | **Self-reported non-adherence** | | |
| --- | --- | --- | --- |
|  | **Adjusted Odds Ratio (95% CI)** | **p-value** | **z score** |
| **Gender** |  |  |  |
| Female | Ref |  |  |
| Male | 1.77 (1.16-2.70) | **0.008** | 2.67 |
| **Adherence self-efficacy** | 0.88 (0.76-1.02) | 0.099 | -1.20 |
| **Task orientated coping** | 0.99 (0.96-1.02) | 0.433 | -1.16 |
| **Emotion orientated coping** | 0.98 (0.94-1.02) | 0.301 | -1.19 |
| **HIV reported stigma** | 1.07 (0.61-1.89) | 0.812 | 0.84 |
| **Variable** | **Pill count <95** | | |
|  | **Adjusted Odds Ratio (95% CI)** | **p-value** |  |
| **Gender** |  |  |  |
| Female | Ref | - |  |
| Male | 1.59 (1.02-2.46) | **0.040** | 2.04 |
| **Household income** | 0.93 (0.88-0.99) | **0.019** | -2.33 |
| **Number of people living together category** | 0.93 (0.87-1.01) | 0.080 | -1.77 |
| **Adherence self-efficacy** | 0.89 (0.78-1.01) | 0.072 | -0.64 |
| **Health literacy** | 0.65 (0.43-0.96) | **0.031** | -1.65 |
| **Household family support** | 1.09 (1.02-1.16) | **0.008** | 1.28 |
| **Task orientated coping** | 0.87 (0.56-1.35) | 0.543 | -2.10 |
| **Variable** | **Virological failure (≥1000 copies/ml)** | | |
|  | **Adjusted Odds Ratio (95% CI)** | **p-value** |  |
| **Gender** |  |  |  |
| Female | Ref | - |  |
| Male | 1.97 (1.14-3.41) | **0.016** | 2.43 |
| **Food insecurity in the last 30 days** |  |  |  |
| No reported food insecurity in the last 30 days | Ref |  |  |
| Reported food insecurity in the last 30 days | 1.64 (0.69-3.91) | 0.266 | 1.28 |
| **Adherence self-efficacy** | 0.99 (0.80-1.22) | 0.898 | 1.27 |
| **Clinician trust** | 0.94 (0.88-1.01) | 0.099 | -1.73 |
| **Coping strategy scores** |  |  |  |
| Task orientated coping | 0.98 (0.94-1.02) | 0.386 | -1.07 |
| Emotion orientated coping | 0.95 (0.90-1.01) | 0.100 | -1.67 |
| **HIV reported stigma** | 0.83 (0.34-2.05) | 0.688 | -0.20 |
| Moderate or severe depressive symptoms | 1.08 (1.01-1.15) | **0.024** | 2.26 |
