# Supplementary material for: Predictors of Treatment Adherence and Virological Failure Among People Living with HIV Receiving Antiretroviral Therapy in a South African Rural Community: A Sub-study of the ITREMA Randomised Clinical Trial
Source: AIDS Behav. 2023 Jun 29;27(12):3863–85. doi: 10.1007/s10461-023-04103-2 (PMC10598166; doi:10.1007/s10461-023-04103-2)
Supplement: Supplementary file 4 — Supplementary file4 (DOCX 25 KB) [file 10461_2023_4103_MOESM4_ESM.docx]

Supplementary material 4: Sensitivity analysis for self-reported adherence: Association of socio-demographic and psycho-social characteristics with ART adherence

|  | **Overall sample, n (%) or median [IQR]** | **Self-reported non-adherence (n=458)** | | | | | |
| --- | --- | --- | --- | --- | --- | --- | --- |
|  |  | **Poor self-reported adherence (n=235, 51.3%)** | **%** | **Good self-reported adherence (n=223, 48.7%)** | **%** | **p-value** | **Chi-square value/ t value** |
| **Sociodemographic characteristics** | | | | | | | |
| **Gender** (male) | 150 (29.9%) | 81 | 34.5 | 53 | 23.8 | **0.012** | 6.3308 |
| **Age** (median) | 42.0 years [36.0-49.0 years] | 42 (37-49) | - | 43 (36-50) | - | 0.735 | 0.3393 |
| **Age** (category) |  |  |  |  |  |  |  |
| <35 years | 114 (22.8) | 49 | 20.85 | 46 | 20.63 |  |  |
| 35-49 years | 257 (51.3) | 128 | 54.47 | 113 | 50.67 |  |  |
| >50 years | 130 (26.0) | 58 | 24.68 | 64 | 28.70 |  |  |
| **Relationship status** (in a relationship) | 294 (58.7) | 139 | 59.15 | 132 | 59.19 | 0.992 | 0.0001 |
| **Education** (secondary/tertiary) | 408 (81.4) | 197 | 81.1 | 176 | 81.9 | 0.828 | 0.8176 |
| **Employment (**unemployed) | 256 (51.1) | 120 | 51.06 | 112 | 50.22 | 0.857 | 0.0323 |
| **Household income median (per 1000.00 ZAR)** (median) | R1600.00 [R700.00-R4200.00] | 1500 (700-4000) |  | 1600 (700-4300) |  | 0.969 | -0.0388 |
| **Household income per month (**category) |  |  |  |  |  |  |  |
| <3,500 ZAR | 346 (69.1) | 167 | 71.06 | 152 | 68.16 |  |  |
| 3,500-10,000 ZAR | 124 (24.8) | 52 | 22.13 | 61 | 27.35 |  |  |
| >10,000 ZAR | 31 (6.2) | 16 | 6.81 | 10 | 4.48 |  |  |
| **Number of people living together** (median) | 5 people [3-7 people] | 5 (3-7) |  | 5 (3-7) |  | **0.095** | 1.6771 |
| **Number of people living together** (category) |  |  |  |  |  |  |  |
| 02-Jan | 58 (11.6) | 29 | 12.34 | 22 | 9.87 |  |  |
| 05-Mar | 233 (46.5) | 111 | 47.23 | 102 | 45.74 |  |  |
| Above 5 | 210 (41.9) | 95 | 40.43 | 99 | 44.39 |  |  |
| **Food insecurity** (in the last 30 days) | 41 (8.2) | 18 | 7.66 | 18 | 8.07 | 0.870 | 0.0268 |
| **Psychosocial characteristics** | | | | | | | |
| **Adherence self-efficacy** (adequate) | 472 (94.2) | 218 | 92.77 | 216 | 96.86 | **0.056** | 3.8641 |
| **Health literacy** (high) | 487 (98.8) | 228 | 98.28 | 218 | 99.09 | 0.457 | 0.5727 |
| **Clinician trust** (high) | 499 (99.6) | 235 | 100.0 | 222 | 99.6 | 0.487 | 1.0564 |
| **Household support** (good) | 434 (86.8) | 205 | 87.23 | 192 | 86.49 | 0.813 | 0.0559 |
| **Non-household family support** (good) | 305 (60.9) | 143 | 60.85 | 135 | 60.54 | 0.945 | 0.0047 |
| **Coping strategy scores** |  |  |  |  |  |  |  |
| **Task-oriented coping** (median) | 26 (21-33) | 26 (20-32) |  | 28 (21-33) |  | **0.029** | 2.2013 |
| **Emotion oriented coping** (median) | 18 (14-22) | 17 (14-21) |  | 18 (14-22) |  | **0.071** | 1.8129 |
| **Avoidance oriented coping** (median) | 15 (12-20) | 16 (12-20) |  | 15 (12-20) |  | 0.653 | -0.4481 |
| **HIV-related (internalized) stigma** (stigma) | 258 (51.8) | 129 | 55.13 | 103 | 46.40 | **0.063** | 3.4754 |
| **Mental Health** (Moderate or severe depressive symptoms) | 31 (6.2) | 16 | 6.84 | 11 | 4.93 | 0.390 | 0.7453 |

Sensitivity analyses: Logistic regression analysis between self-reported non-adherence and socio-demographic and pyscho-social characteristics of ART participants in the ITREMA trial.

| **Variable** | **Self-reported adherence difficulties** | | |  |
| --- | --- | --- | --- | --- |
|  | **Adjusted Odds Ratio (95% CI)** | **p-value** | **z value** |  |
|  |  |  |  |  |
|  |  |  |  |  |
| **Gender** |  |  |  |  |
| Female | Ref |  |  |  |
| Male | 1.69 (1.10-2.55) | **0.017** | **2.40** |  |
| Number of people living together (median) | 0.95 (0.89-1.02) | 0.147 | -1.45 |  |
| **Adherence self-efficacy** |  |  |  |  |
| Inadequate adherence self-efficacy | Ref |  |  |  |
| Adequate adherence self-efficacy | 0.55 (0.22-1.39) | 0.205 | -1.27 |  |
| **Coping strategy scores (CISS)** |  |  |  |  |
| Task-oriented coping | 0.98 (0.95-1.01) | 0.106 | -1.62 |  |
| Emotion-oriented coping | 0.97 (0.94-1.01) | 0.162 | -1.40 |  |
| **HIV related stigma** |  |  |  |  |
| No reported stigma | Ref |  |  |  |
| Reported stigma | 1.17 (0.78-1.74) | 0.443 | 0.77 |  |
